# Chemistry of zipping reactions in mesoporous carbon consisting of minimally stacked graphene layers†

**DOI:** 10.1039/d3sc02163g

**Published:** 2023-07-18

**Authors:** Tian Xia, Takeharu Yoshii, Keita Nomura, Keigo Wakabayashi, Zheng-Ze Pan, Takafumi Ishii, Hideki Tanaka, Takashi Mashio, Jin Miyawaki, Toshiya Otomo, Kazutaka Ikeda, Yohei Sato, Masami Terauchi, Takashi Kyotani, Hirotomo Nishihara

**Affiliations:** a Institute of Multidisciplinary Research for Advanced Materials, Tohoku University 2-1-1 Katahira, Aoba-ku Sendai Miyagi 980-8577 Japan takeharu.yoshii.b3@tohoku.ac.jp hirotomo.nishihara.b1@tohoku.ac.jp; b Advanced Institute for Materials Research (WPI-AIMR), Tohoku University 2-1-1 Katahira, Aobaku Sendai Miyagi 980-8577 Japan; c International Research and Education Center for Element Science Faculty of Science and Technology, Gunma University 1-5-1 Tenjincho Kiryu Gunma 376-8515 Japan; d Research Initiative for Supra-Materials (RISM), Shinshu University 4-17-1 Wakasato Nagano 380-8553 Japan; e Interdisciplinary Graduate School of Engineering Sciences, Kyushu University 6-1 Kasuga-koen Kasuga Fukuoka 816-8580 Japan; f Institute for Materials Chemistry and Engineering, Kyushu University 6-1 Kasuga-koen Kasuga Fukuoka 816-8580 Japan; g Institute of Materials Structure Science, High Energy Accelerator Research Organization (KEK) 203-1 Shirakata Tokai Ibaraki 319-1106 Japan; h J-PARC Center, High Energy Accelerator Research Organization (KEK) 2-4 Shirakata-Shirane Tokai Ibaraki 319-1106 Japan; i School of High Energy Accelerator Science, The Graduate University for Advanced Studies 203-1 Shirakata Tokai Ibaraki 319-1106 Japan; j Graduate School of Science and Engineering, Ibaraki University 162-1 Shirakata Tokai Ibaraki 319-1106 Japan

## Abstract

The structural evolution of highly mesoporous templated carbons is examined from temperatures of 1173 to 2873 K to elucidate the optimal conditions for facilitating graphene-zipping reactions whilst minimizing graphene stacking processes. Mesoporous carbons comprising a few-layer graphene wall display excellent thermal stability up to 2073 K coupled with a nanoporous structure and three-dimensional framework. Nevertheless, advanced temperature-programmed desorption (TPD), X-ray diffraction, and Raman spectroscopy show graphene-zipping reactions occur at temperatures between 1173 and 1873 K. TPD analysis estimates zipping reactions lead to a 1100 fold increase in the average graphene-domain, affording the structure a superior chemical stability, electrochemical stability, and electrical conductivity, while increasing the bulk modulus of the framework. At above 2073 K, the carbon framework shows a loss of porosity due to the development of graphene-stacking structures. Thus, a temperature range between 1873 and 2073 K is preferable to balance the developed graphene domain size and high porosity. Utilizing a neutron pair distribution function and soft X-ray emission spectra, we prove that these highly mesoporous carbons already consist of a well-developed sp^2^-carbon network, and the property evolution is governed by the changes in the edge sites and stacked structures.

## Abstract 2

The progress of graphene-zipping reactions in graphene-based materials is successfully visualized using advanced temperature-programmed desorption (TPD) up to 2073 K.

## Graphical abstract

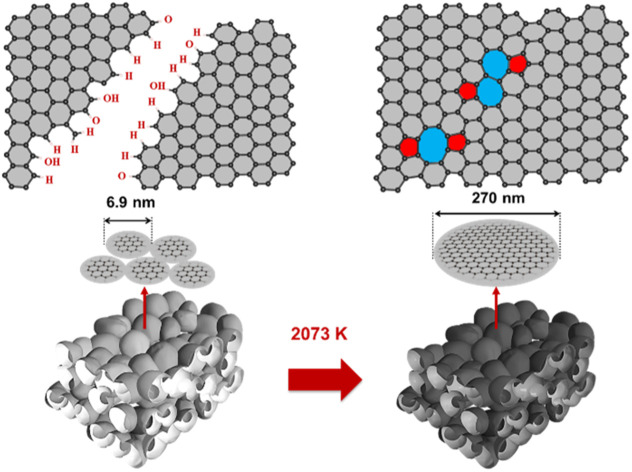

## Introduction

Porous carbon materials have attracted increasing attention in various fields due to their unique structure and properties.^1^ Their high specific surface area and superior electrical properties make them suitable for a diverse number of applications, such as energy storage,^2,3^ catalysis,^4^ and environmental remediation.^5^ Most porous carbons are typically synthesized at a temperature range between 873 and 1273 K. For example, traditional porous carbons, like activated carbons, are synthesized by carbonization of an organic precursor below 1273 K, followed by chemical or physical activation at 873–1173 K.^6,7^ Templated nanoporous carbons are generally synthesized by carbonization of the mixture of organic precursor and an inorganic template, typically below 1273 K, followed by template removal.^8^ Furthermore, post-synthesis heat treatment (HT) of porous carbons has proved popular to remove specific types of oxygen-functional groups^9,10^ and improve the degree of graphitization of porous carbons.^3,11,12^ HT above 1273 K is especially crucial to remove graphene-edge sites *via* zipping reactions and to achieve advanced carbon materials with high durability and electrical conductivity,^13^ whereas HT above 1273 K often causes pore shrinkage due to graphitization.^14–16^

**Fig. 1:**
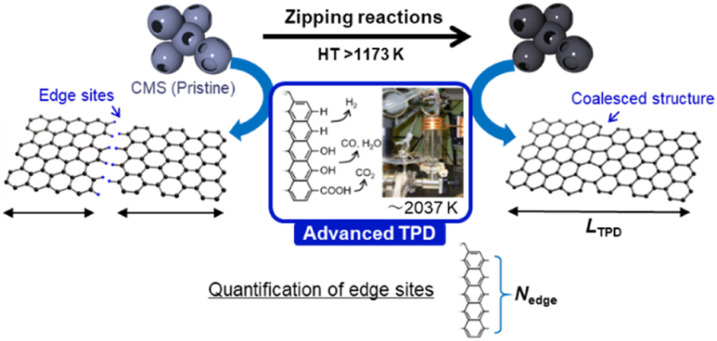
Schematic of HT of CMS and the acommpanied graphene-zipping reactions. The advanced temperature-programmed desorption (TPD) is used to quantify the carbon edge sites and calculate the average graphene-domain size (*L*_TPD_).

Recently, highly mesoporous carbons consisting of a few-layer graphene wall have attracted interest for various applications, including supercapacitors,^17^ high-capacity adsorbents,^18^ new types of heat pumps,^19^ lithium-sulfur batteries,^20^ catalyst supports,^21^ polymer-electrolyte fuel cells,^22^ and lithium-oxygen batteries.^23,24^ It has been reported that such specific mesoporous carbons show almost no structural change up to 2073 K, where graphene-zipping reactions take place to improve the electric conductivity and chemical stability of the carbon framework.^13^ Thus, in this work we focus on the structural change of such a unique mesoporous carbon, carbon mesosponge (CMS), between 1173 K and 2873 K. Since CMS mesoporosity remains almost intact up to 2073 K,^13^ it is an interesting model system for analyzing graphene-zipping reactions and the resultant increase in graphene domain size at such high-temperatures (Fig. 1). It is also essential to elucidate the optimal conditions for facilitating graphene-zipping reactions without the development of graphene stacking to synthesize advanced functional carbon materials like graphene mesosponge (GMS), that are expected to show promise in a variety of applications due to its developed mesoporosity, high specific surface area, high electrical conductivity, high durability, and unique mechanical flexibility.^13^

Herein, the structural evolution of CMS is thoroughly analyzed by various methods, including transmission electron microscopy (TEM), N_2_ gas adsorption, powder X-ray diffraction (PXRD), Raman spectroscopy, advanced temperature-programmed desorption (TPD) up to 2073 K (Fig. 1), neutron scattering, H_2_O-vapor adsorption, solid-state ^13^C-NMR, soft X-ray emission spectroscopy (SXES), and magnetic susceptibility measurement using a superconducting quantum interference device (SQUID). Moreover, structural properties relationships are examined, focusing on their thermal stability in the air, electrochemical stability, elasticity, and electric conductivity. This study delves into a profound comprehension of the intricate molecular-level structural transformations that transpire during the process of zippering reactions amid graphene sheets at a temperature range from 1273 K to 2273 K. The findings furnish a roadmap towards attaining coveted attributes in ubiquitous porous carbon materials and other carbonaceous frameworks *via* HT, thereby propelling the advancement of superior porous carbon materials.

## Experimental

### Sample preparation

Highly mesoporous carbon CMS was prepared following the method we have previously reported.^13^ About 3 g of γ-Al_2_O_3_ nanoparticles (Al_2_O_3_-NP; TM-300, Taimei Chemicals Co., Ltd.) with an average particle size of 7 nm was mixed with 10 g of quartz sand (Wako Pure Chemical Industries, Ltd.) as a spacer. The mixed powder was placed into a homemade quartz tube, and chemical vapor deposition (CVD) (20 vol%-methane in Ar, 1173 K for 2 h) was performed. We previously reported the optimal conditions for the deposition of almost single-layer graphene sheets onto Al_2_O_3_ based on the unique catalysis of oxygen-vacancy sites generated at the initial stage of CVD.^25^ After cooling to room temperature, the sample was sieved to separate the carbon-coated Al_2_O_3_-NP and the quartz sand. Finally, CMS was obtained by chemically etching the carbon-coated Al_2_O_3_-NP with HF for 6 h at room temperature. Then, the CMS was annealed at designated temperatures between 1173 and 2873 K for 1 h. The HT was performed in a vacuum below 2073 K and in Ar above 2073 K. The heat-treated samples are denoted by the HT temperature (K). Note that 2273, 2573 and 2873 samples for TPD, SXES, electrochemical stability, elasticity, and electric conductivity measurements were prepared with an annealing time of 30 min.

### Characterization

The nanostructures of the samples were observed with a transmission electron microscope (TEM: JEM-2010, JEOL Ltd.) with an accelerating voltage of 200 V. N_2_ adsorption measurements were carried out at 77 K using a volumetric sorption analyzer (BEL Japan, BELSORP MAX). The specific surface area (*S*_BET_) was calculated by the Brunauer–Emmett–Teller (BET) method in the pressure range of *P*/*P*_0_ = 0.1–0.3. PXRD patterns of the samples were recorded by using an X-ray diffractometer (MiniFlex600, Rigaku Co.) with CuKα radiation generated at 40 kV and 15 mA. The average size of the planar graphene moiety (*L*_PXRD_) detected by carbon 10 peak is calculated using the Scherrer equation:1
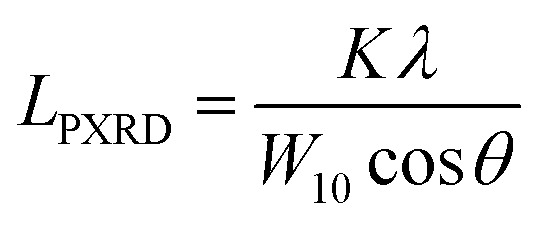
where *K* is the Scherrer constant (taken as 1.84), *λ* is the X-ray wavelength (0.15418 nm here), *W*_10_ is the FWHM of the diffraction peak of the carbon (10) plane, and *θ* is the diffraction angle. The intensity was normalized using the peak at around 43° assigned to the carbon (10) plane. Raman spectra were measured with a Jasco NRS-3300FL spectrometer with the 532.2 nm line, and the intensity was normalized using the G-band. To estimate the number of edge sites, a TPD measurement was performed up to 2073 K with a heating rate of 10 K min^−1^ using the advanced TPD system developed by our group (Fig. 1).^26,27^ The thermal decomposition of oxygen-functional groups and hydrogen-terminated edge sites generates CO, CO_2_, H_2_O, and H_2_. The number of the terminated edge-sites (*N*_edge_ [mmol g^−1^]) can be estimated from the amount of the desorbed gas as

2 *N*_edge_ = *N*_CO_ + *N*_CO_2__+*N*_H_2_O_ (>673 K) + 2N_H_2__

where *N*_CO_, *N*_CO_2__, *N*_H_2_O_, and *N*_H_2__ are the amount of desorbed gas of CO, CO_2_, H_2_O, and H_2_, respectively.^28^ Note that the number of H-terminated edge sites was calculated by doubling the amount of H_2_ gas emitted since one molecule of H_2_ is produced from two H-terminated edge sites. The interpretation of CO, CO_2_, and H_2_O is actually complicated. For example, when a phenol group is decomposed as CO, a H-terminated edge site is left, and it is desorbed as H_2_ by coupling with H desorbed from another H-terminated edge site. Thus, it causes an overestimation. On the other hand, if H_2_O is formed from H-terminal edge sites and phenols, *N*_edge_ could be underestimated since two edge sites count as one. Since it is difficult to distinguish all the diverse reactions, we use the simplified eqn (2) in this work. Subsequently, the average size of the graphene layers (*L*_TPD_ [nm]) can be estimated, where we assume that the shape of graphene sheets is coronene-type hexagonal as3
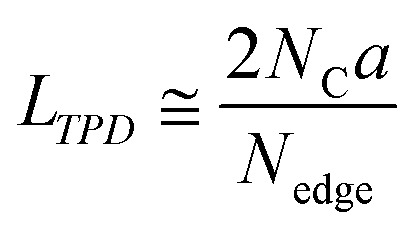
where *a* and *N*_C_ correspond to the lattice parameter of the *a*-axis (0.2461 nm) and the number of carbon atoms (approximately 83.26 mmol g^−1^ when the size of the graphene sheet is much larger than *a*), respectively.^26,28^ To discuss the presence of carbon polygons and curvature in carbon frameworks, a high-resolution-pair distribution function (PDF), *g*(*r*), was obtained using the neutron total scattering instrument, NOVA (BL21), at the Material and Life Science Facility of the Japan Proton Accelerator Research Complex. The structure factor, *S*(*Q*), was obtained by irradiating 100 mg of powder sealed in a V–Ni null scattering sample container for 4.5 h at room temperature with a 500 kW spallation neutron source and correcting the obtained diffraction profile for the background, self-attenuation, number of incident neutrons, solid angle of detectors, multiple scattering, and incoherent scattering cross-sections. *g*(*r*) was derived by Fourier transformation of *S*(*Q*) in the *Q* range of 0.2–55.0 Å. The adsorption isotherms of H_2_O vapor were measured at 298 K using a volumetric sorption analyzer (BEL Japan, BELSORP MAX). ^13^C cross polymerization-magic angle spinning (CP-MAS) NMR spectra were obtained on a JEOL JNM-ECA800 (800 MHz) spectrometer. SXES was performed on an original SXES attached to a scanning electron microscope of JSM-6480LV.^29,30^ A varied-line-spacing grating, JS200N, with an average groove density of 1200 lines per mm, was used in these experiments. Because the acceptable energy range of this spectrometer is limited, the second-order spectrum of C K-emission, C–K(2), was measured. The spin density in each carbon sample was calculated from its magnetic susceptibility.^26,31^ The measurement was performed with a SQUID magnetometer (MPMS-5SW, Quantum Design Inc.) under a field strength of 1 T between 2 K and 300 K. The observed susceptibility *χ* is expressed by using the Curie–Weiss term *χ*_C_ and temperature-independent term *χ*_0_ as4
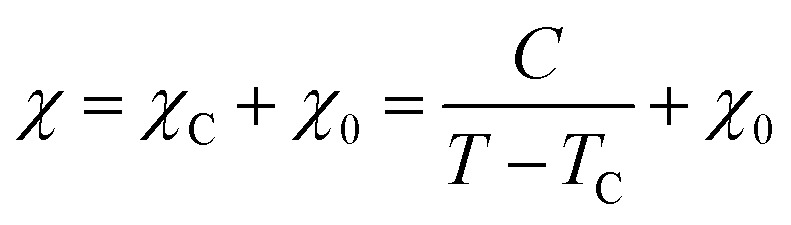
where *C*, *T*, and *T*_C_ are the Curie constant, temperature, and Weiss temperature, respectively. Eqn (4) can be transformed into5
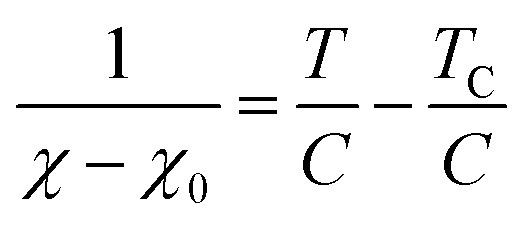


To determine *C*, 1/(*χ* − *χ*_0_) was plotted against *T*. A suitable range of *T* was selected so that the 1/(*χ* − *χ*_0_) *vs. T* curve exhibits a linear relationship. The temperature-independent term *χ*_0_ was determined to maximize the coefficient of determination (*R*^2^) of the fitted line. Note that the plots were performed in a narrow temperature range below 20 K for the samples after HT because temperature-dependent Landau diamagnetism was observed. Then, the spin density *N*_spin_ was calculated using *C* obtained from eqn (5), according to6
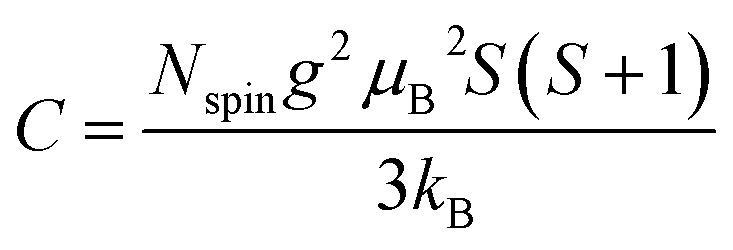
where *g*, *μ*_B_, *S*, and *k*_B_ are the *g*-factor (assumed to be 2.002319), the Bohr magneton (= 9.274× 10^−24^ J T^−1^), the total spin quantum number, and Boltzmann constant (= 1.381 × 10^−23^ J K^−1^), respectively. *S* can take the value of 1/2 or 1 for unpaired electron spins. Here, *N*_spin_ is calculated assuming *S* is 1/2 for simplicity.^26^

### Theoretical simulations of zipping reactions

2946 carbon atoms were randomly placed on the surface of a hypothetical spherical alumina of 4.66 nm in diameter using Monte Carlo simulation. Then, a quench molecular dynamics (QMD) simulation was performed to investigate the mechanism of carbon–carbon interactions on the surface of the spherical alumina (self-made simulation code). The reaction state summation^32^ potential was applied for the interaction between carbon atoms and the Lennard Jones (LJ) potential for the interaction between carbon atoms and the alumina surface. The alumina surface structure was assumed to be a spherical shell with a uniform atomic density. To prevent the desorption of carbon atoms from the alumina surface during the QMD simulation, the energy parameter of the LJ interaction potential between solid atoms and carbon atoms was set to *e*/*k* = 70 000 K (*k* is the Boltzmann constant and the size parameter was *s* = 0.34 nm). The initial temperature was 10 000 K, and the system was quenched to 298 K during the QMD simulation using the velocity rescaling method (quench rate: 13 K ps^−1^). The equations of motion were integrated using the velocity Verlet method with a time step of 0.05 fs for 750 ps. Finally, the CMS model was obtained by deleting the alumina sphere and all carbon atoms with a coordination number of one from the spherical carbon.

We created a rift in the graphene wall of the CMS model, and the generated edges were terminated by hydrogen and oxygen-functional groups. Specifically, 4 ether, 11 hydroxyl, 20 methine, 10 carboxyl, and 4 carbonyl, were attached leading to a total of 2589 atoms present in the CMS model. The molecular dynamics (MD) simulation was performed using Large-scale Atomic/Molecular Massively Parallel Simulator (LAMMPS)^33^ and a ReaxFF force field^34^ was employed for the geometry evolution of the CMS structure where the temperature increased from 10 K to 298 K at a rate of 230 K ps^−1^, and a time step of 0.125 fs. The simulation cell was 7.0 nm × 7.0 nm × 7.0 nm in size. After increasing the temperature from 298 K to 3000 K (17 K ps^−1^), the canonical ensemble MD simulation (NVT-MD) was performed for 125 ps while the temperature was controlled at 3000 K using the Nose–Hoover method. In the final step, gas molecules leaving the CMS model were removed from the simulation cell. Then, the NVT-MD simulation was run for 250 ps at 3000 K, and again gas molecules were removed from the simulation cell in the final step. This was repeated twice. The temperature was then controlled at 3000 K for 125 ps and then lowered to 298 K (17 K ps^−1^) to generate the CMS model with partially fused rifts.

### Evaluation of electrochemical and mechanical properties

Air-oxidation durability was examined using a thermogravimetry analyzer (Shimadzu TGA-51). The samples were heated up to 1173 K for 180 min under air flow. Electrochemical oxidation resistance was also examined by cyclic voltammetry (CV) using a three-electrode cell at 298 K. The mesoporous carbon samples were mixed with a binder polymer (PTFE; PTFE 6-J, Du Pont-Mitsui Fluorochemicals Co. Ltd.) and carbon black (Denka black, Denki Kagaku Kogyo Kabushiki Kaisha) at a ratio of 90 : 5 : 5. The resulting mixture was molded into a square sheet (1 × 1 cm^2^, 5–10 mg), and sandwiched between a Pt mesh, to form a working electrode. The electrolyte was 1 M Et_4_NBF_4_/PC. An activated carbon fiber (Unitika, A20) was used for the counter electrode, which was prepared in the same method as working electrodes except for its amount (*ca.* 20 mg). The reference electrode was Ag/AgClO_4_. The CV was performed as follows. First, the CV scans were performed in the potential range of −0.5 to 0.5 V with a scan rate of 1 mV s^−1^, and then the upper-limit potential was gradually increased by 0.1 V up to 2.1 V. In each potential range, the CV scan was repeated four times. The irreversible electric charge (*Q*_ir_) was calculated as

7 *Q*_ir_ = *Q*_positive_ − *Q*_negative_

where *Q*_positive_ and *Q*_negative_ are the total charges during a positive-direction scan above an open circuit potential and the one during a negative-direction scan, respectively.^35^ The bulk modulus of samples was measured from a mercury intrusion test using an Autopore IV 9510 (Micromeritics Instrument Corporation). The stress–strain curve of a powdery sample can be obtained from mercury intrusion by choosing a relatively low Hg pressure region at which the nanopores are not yet impregnated by Hg.^13,19^ The bulk modulus is estimated from the slope of the curve according to the following equation.8
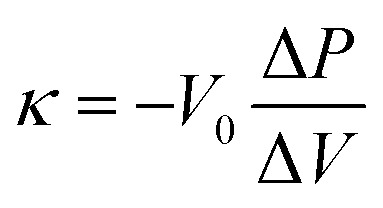
where *κ*, *V*_0_, Δ*P*, Δ*V* are the bulk modulus, the initial volume, the changes of pressure, and the changes of volume, respectively. The electric conductivity of the carbon powders was measured by a two-probe method.^13^ The conductivity was measured while applying pressure to the powdery sample up to 90 MPa. At the same time, the change in the density of the sample bed was measured.

## Results and discussion

### Morphological change by HT

**Fig. 2:**
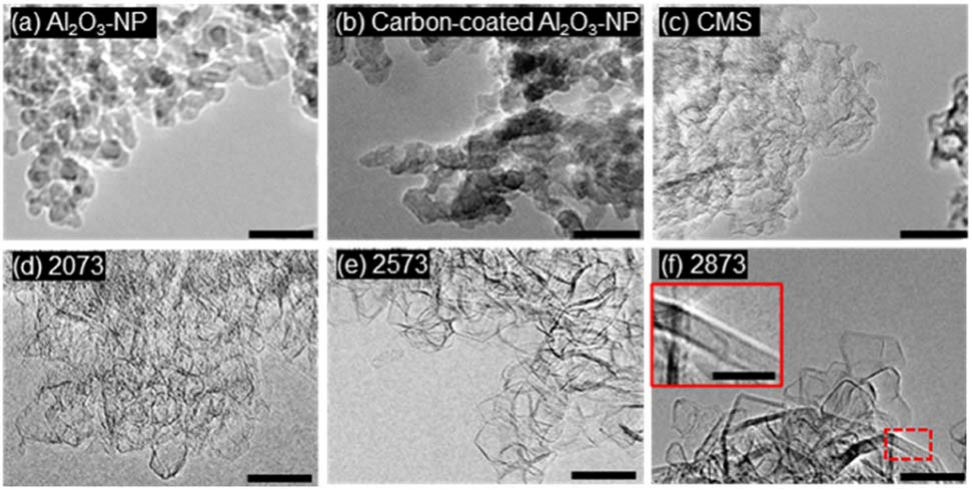
TEM images of (a) Al_2_O_3_, (b) carbon-coated Al_2_O_3_, (c) CMS, and annealed samples at (d) 2073 K, (e) 2573 K, and (f) 2873 K. The scale bar is 20 nm. The highly stacked graphene structure in (f) was highlighted and magnified (the scale bar in magnified part is 10 nm).

Each primary particle of the Al_2_O_3_-NP template was about 7 nm in diameter and partially connected (Fig. 2a). The size of secondary particles is around 30–100 μm (the data are not shown here). Al_2_O_3_-NP has unique catalytic properties for the conversion of methane into graphene sheets, and the formation of the first layer occurs about three times faster than the following layers.^25,36,37^ Thus, 1–2 layers of carbon sheets can be formed on the surface of the Al_2_O_3_-NP (Fig. 2b). The shape of the templated carbon (CMS) roughly imitates the morphology of the Al_2_O_3_-NP (Fig. 2c), with primary cells composed of curved graphene walls bonded to each other. The size of the primary cells of CMS does not change even after HT at 2073 K (Fig. 2d), but becomes larger at higher temperatures (Fig. 2e and f). Although the change of graphene stacking number is difficult to see in Fig. 2e because of the limitation of the TEM resolution, the number of carbon layers increases after HT above 2073 K, as shown later. CMS comprises of curved graphene walls, and the structure is retained up to 2073 K. These results indicate that HT below 2073 K does not significantly change the cellular graphene structure, while the significant rearrangement of carbon atoms occurs above 2073 K to form stacked graphene walls with larger cellular structures because of the progress of the graphitization.^38^

**Fig. 3:**
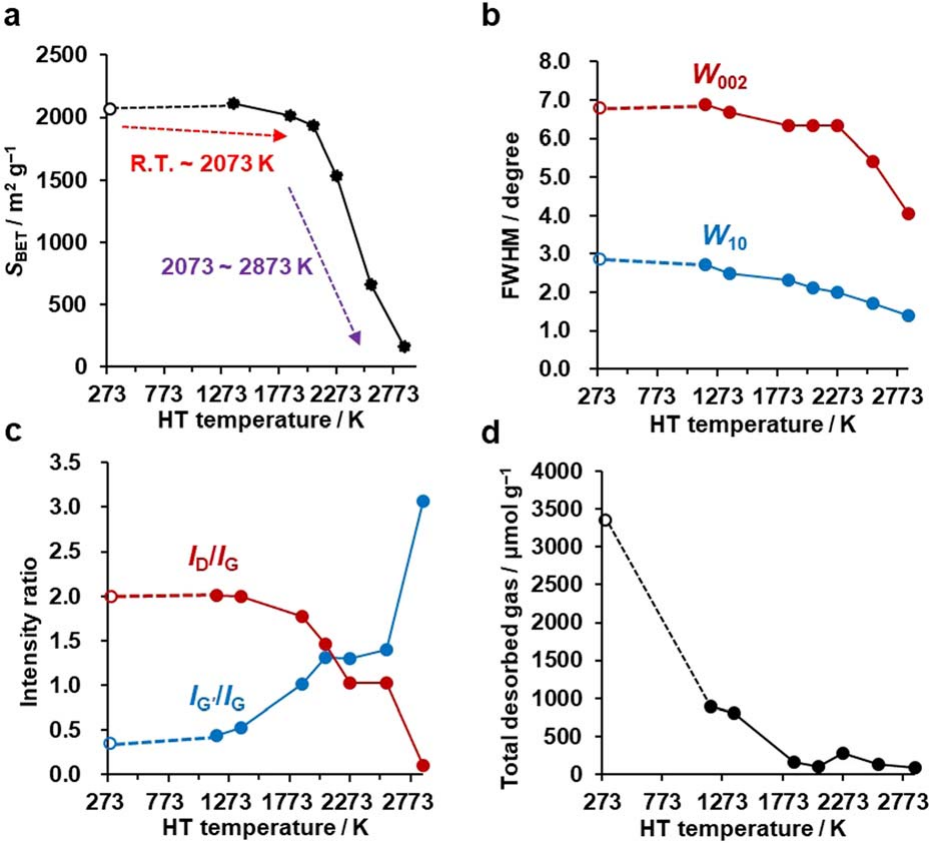
The evolution of pore structure, crystallinity, and edge-structure of CMS upon HT. CMS is plotted at 298 K as a reference. (a) Plot of *S*_BET_ calculated from N_2_ adsorption isotherms *versus* HT temperature. (b) Evolution of *W*_002_ and *W*_10_ calculated from PXRD as a function of HT temperature. (c) Variation of *I*_D_/*I*_G_ and *I*_G′_/*I*_G_ measured by Raman spectroscopy plotted *versus* HT temperature. (d) Total desorbed amount of gas measured by advanced TPD *versus* HT temperature.

N_2_ adsorption–desorption isotherms are shown in Fig. S1,† and the BET specific surface areas (*S*_BET_) are plotted against the HT temperature in Fig. 3a. It should be noted that we plot the as-synthesized CMS data provisionally at room temperature (298 K) as a reference. Although the synthesis process of CMS includes CVD at 1173 K, the CVD-derived carbon is in a metastable state because of the protective effect of the inorganic template. After template removal, a structural change occurs, including the introduction of a significant number of oxygen-functional groups.^39^ Thus, the thermal stability of templated carbons is generally lower than expected from their CVD temperatures. There is no significant change in *S*_BET_ up to 2073 K. Then, *S*_BET_ decreases rapidly when the annealing temperature reaches 2273–2873 K, suggesting the mesoporous structure's collapse, as indicated by the TEM observations (Fig. 2e and f). The average stacking number of graphene sheets can be further estimated based on the *S*_BET_, where the measured *S*_BET_ value is divided by the theoretical specific surface area of graphene of 2627 m^2^ g^−1^.^13^ The average stacking numbers of CMS before and after HT at 2073 K are similar at 1.3 and 1.4, respectively. However, the number increases significantly to 16.4 layers at 2873 K. These results are also consistent with the TEM observations (Fig. 2). In the N_2_ adsorption/desorption isotherms in Fig. S1b,† CMS and its heat-treated samples show clear hysteresis loops even after annealing at 2873 K, indicating the formation of connected mesopores.

### Crystalline change by HT

PXRD was used to evaluate the crystallinity of graphene sheets (Fig. S2a†).^40,41^ The peak at around 25° is assigned to the carbon (002) plane, corresponding to the stacking structure of graphene layers. The peak at around 43° is assigned to the carbon (10) plane, corresponding to the in-plane diffraction of graphene, typically found in turbostratic carbons.^42^ Interestingly, no significant change is observed in the PXRD pattern up to 2273 K, while there is a noticeable increase in the 002 peak intensity above 2573 K, indicating the development of stacked graphene-sheets. Next, the full width at half maximum (FWHM) of 002 (*W*_002_) and 10 (*W*_10_) peaks are used as a measure for the degree of graphene stacking and the domain size of the graphene basal plane, respectively (Fig. 3b). Specifically, the larger the *W*_002_, the lower the degree of graphene stacking, and the larger the *W*_10_, the smaller the size of the basal plane. The generally observed trend is that *W*_002_ and *W*_10_ decrease as a function of HT temperature, indicating an increase in graphene stacking and domain size. *W*_002_ shows a rapid decrease above 2273 K, as seen from the intensive development of the carbon 002 peak in Fig. S2a.†^43^ The gradual reduction in *W*_10_ indicates a successive growth of the basal plane over the whole temperature range. Notably, from Fig. S2a,† we see that the carbon 002 and 10 peaks become asymmetric above 2573 K. This could be due to the broad and sharp peaks overlapping, suggesting that part of the carbon framework inhomogeneously turns into a highly crystalline structure. These PXRD results agree with the above structural discussion based on the TEM images and N_2_ adsorption isotherms.

Next, Raman spectroscopy was performed to analyze the degree of disorder and defects in the graphene framework (Fig. S2b†). Here, three peaks were observed in the samples, *i.e.*, the D-, G-, and G′- (also known as 2D) bands.^44–46^ The D-band stands for a breathing mode of a carbon hexagon, which becomes active at carbon defects.^47^ G-band and G′-band are the characteristic peaks of the graphene structure. The position of the G′-band is sensitive to the number of layers and the stacking arrangement of the graphene layers. In Fig. S2b,† the intensity of the D-band decreases with increasing the HT temperature, indicating fewer defects after high-temperature HT. The intensity ratio of the D-band and G-band (*I*_D_/*I*_G_) is plotted in Fig. 3c. A higher *I*_D_/*I*_G_ ratio generally indicates a higher concentration of defects or structural disorders in the material.^48^ Notably, the *I*_D_/*I*_G_ decreases between 1373 and 2073 K, at which nano-level morphology is still not remarkably changed, as found from TEM (Fig. 2) and N_2_ adsorption (Fig. 3a). This suggests the reduction of the microscopic defects that are not detected by TEM and N_2_ adsorption in this temperature range. The *I*_D_/*I*_G_ further decreases above 2073 K because of the development of planar and stacked graphene structures, as suggested by TEM (Fig. 2) and PXRD (Fig. 3b and S2a†). PXRD shows relatively broad carbon 002 and 10 peaks even at 2873 K, indicating that CMS can be categorized as a hardly graphitizable carbon (hard carbon). In conventional hard carbons, most edge sites are removed at 2073 K,^26^ but graphitization is prohibited by a rigid carbon framework consisting of stacked and curved structures even above 2773 K. Thus, hard carbons heat-treated above 2773 K still show intense D-band, because of the significant curvature.^49,50^ However, the *I*_D_/*I*_G_ ratio of CMS becomes almost zero at 2873 K. This means that the unique framework structure of CMS, *i.e.*, rarely stacked graphene sheets, enhances the rearrangement of carbon atoms, and both carbon edge sites and topological defects are significantly decreased at 2873 K. Also, the upshift of the G′-band above 2073 K indicates carbon-atom rearrangement to form a stacked graphene structure.^44^ The *I*_G′_/*I*_G_ ratio gradually increases with increasing HT temperature but should be discussed separately before and after the development of graphene stacking. Before the occurrence of significant graphene stacking below 2073 K, the increase in *I*_G′_/*I*_G_ is attributed to zipping reactions between small graphene sheets to form better quality single-graphene structures (a detailed discussion on edge fusion is given later). As discussed, the graphene-stacking structures are developed above 2073 K; therefore, the *I*_G′_/*I*_G_ is expected to decrease.^51^ However, the *I*_G′_/*I*_G_ does not fall above 2073 K and rapidly increases at 2873 K in Fig. 3c. This is possibly because of the formation of specific stacking angles giving an intense G′ peak.^52,53^ The formation of turbostratic structures is also supported by the PXRD results (Fig. S2a†).

### Edge-structure change by HT

**Fig. 4:**
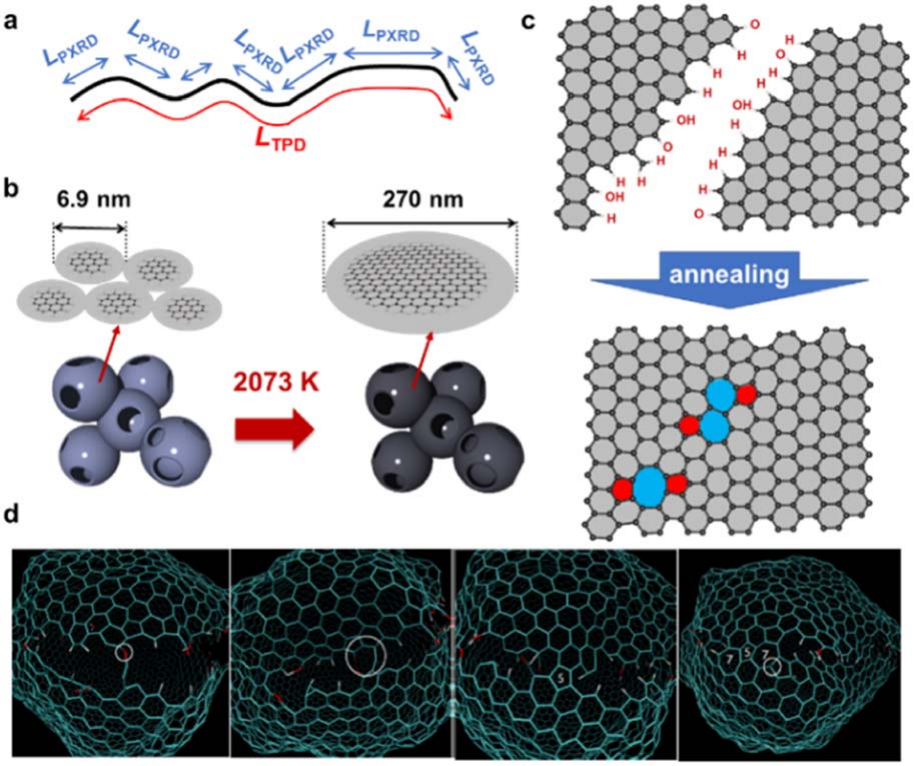
(a) An illustration of and comparison of *L*_PXRD_ and *L*_TPD_. (b) Evolution of the entire graphene size before and after 2073 K. (c) An illustration of zipping reaction of graphene domains. (d) Molecular dynamic simulation of the zipping reactions.

An advanced TPD analysis was carried out to quantify the number of edge sites in the samples. Our TPD system can reach a temperature of 2073 K, which is necessary to determine the total gas evolution of H_2_, H_2_O, CO, and CO_2_ from oxygen functional groups and hydrogen-terminated edge sites in carbon materials.^26^ Fig. S3† shows each amount of desorbed gas from CMS and its heat-treated samples (see also Fig. S4† for the gas-evolution profiles). CMS is synthesized by a hard template method, and many oxygen-functional groups are introduced upon template removal.^39^ Therefore, CMS emits a large amount of gases. H_2_ gas emission occurs in two steps, before and after 1073 K (Fig. S4a†). The former is ascribed to the H_2_ emission along with the decomposition of H-including oxygen-functional groups such as –COOH from the intense H_2_O, CO, and CO_2_ emissions below 1073 K (Fig. S4b–d†). The latter is ascribed to graphene-zipping reactions.^24^ As shown in Fig. 3d, the total amount of desorbed gas significantly decreases at temperatures below 1873 K, indicating the progress of the graphene zipping reactions,^24^ and then remains relatively low level up to 2873 K. Here, the average graphene-domain size (*L*_TPD_) can be estimated using the total number of edge sites analyzed by the TPD method (see Experimental section for calculation details).^26^ While *L*_PXRD_ represents the size of the planar graphene moiety in a curved graphene sheet, *L*_TPD_ can represent the entire graphene size regardless of the curvature (Fig. 4a). Table S1† summarizes *L*_PXRD_ and *L*_TPD_ of CMS and its heat-treated samples. *L*_TPD_ shows a significant change even below 2073 K, unlike *L*_PXRD_. *L*_TPD_ increases 39 times after annealing at 2073 K (from 6.9 nm to 270 nm). This means that approximately 1100 graphene fragments (size: 6.9 nm) coalesce to form a larger structure *via* zipping reactions. Thus, the material synthesized at 2073 K (called GMS) comprises of a continuous edge-free curved graphene sheet with nano-cellular structures (Fig. 2d). The evolution of the graphene size is estimated by TPD and depicted in Fig. 4b. *L*_TPD_ stays almost constant from 2073 K to 2873 K, indicating that the zipping reactions are terminated at 2073 K and drastic rearrangement forming graphene stacking structures begins above this temperature. Interestingly, the total gas evolution amount slightly increases from 2073 K to 2273 K. This could be ascribed to the generation of some dangling bonds along with the carbon atom rearrangement forming stacking structures at 2273 K, as suggested by the decrease of *S*_BET_ (see Fig. 3a). The dangling bonds formed during HT react with O_2_ and H_2_O when the sample is exposed to air after HT, and TPD detects some gas evolution.

The zipping reactions between the graphene sheets are further discussed below. TPD results (Fig. 3d) indicate that the zipping reactions are almost complete at 1873 K. The Raman D-band is derived not only from edge sites but also from carbon 5- and 7-membered rings known as topological defects.^24^ Thus, even after zipping reactions, the Raman spectrum still shows a relatively large *I*_D_/*I*_G_ at 1873 K (Fig. 3c). Indeed, we have recently reported the origin of an intense D-band of GMS is derived from topological defects by atomic-resolution TEM and theoretical calculations.^24^Fig. 4c shows an illustration of the progress of a zipping reaction. A decrease of the *I*_D_/*I*_G_ from 1873 K to 2073 K suggests the partial rearrangement of topological defects into carbon hexagons because the total TPD gas evolution does not significantly decrease. From 2073 K to 2873 K, *I*_D_/*I*_G_ successively decreases, indicating a further transformation of topological defects into carbon hexagons. Such structural change accords with the results of TEM (Fig. 2d–f) and PXRD (Fig. 3b and S2a†).

The zipping reactions of the edge sites were further investigated using molecular dynamics simulations. As shown in Fig. 4d, the carbon edge sites gradually fuse through the zipping reactions. As a result, topological defects of 5- and 7-membered rings are introduced throughout the boundary. These results support our experimental conclusions indicating topological defects are indeed formed by graphene zipping reactions.^24^

**Fig. 5:**
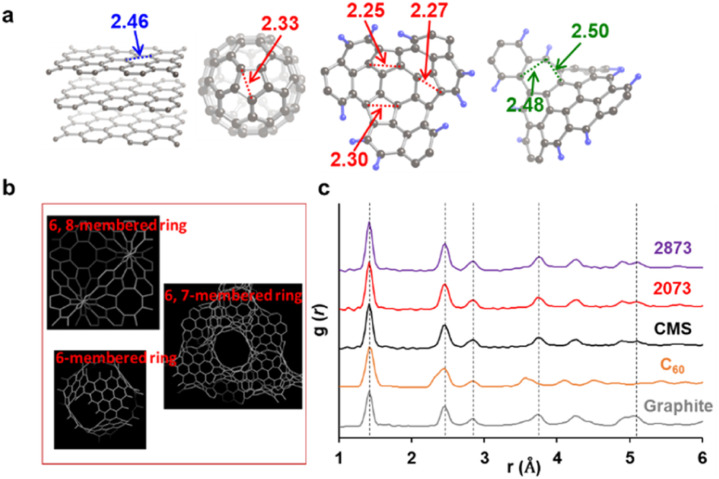
(a) Distances to the second nearest neighbor carbon atoms in different carbon frameworks. (b) A schematic of possible carbon frameworks of GMS. (c) PDF results of CMS, its heat-treated samples, and reference carbon materials (graphite and C_60_). The peak positions at the 1st, 2nd, 3rd, 4th, and 7th neighbor carbon atoms in graphite are shown with vertical dashed lines.

When there is a significant amount of carbon 5- and 7-membered rings, they can be detected by high-resolution-pair distribution function (PDF) obtained from neutron diffraction.^54^ The second nearest neighbor carbon distance are 2.33, 2.46, and 2.48–2.50 Å in carbon pentagons, hexagons, and heptagons, respectively (Fig. 5a).^54^ It was theoretically predicted that structures containing 19% pentagons and 23% heptagons show the corresponding resolve peaks, respectively.^54^ CMS and its heat-treated samples have nano-cellular structures, and should include carbon non-hexagon rings at their curvatures (Fig. 5b). The PDF patterns of these samples are shown in Fig. 5c, together with reference data from graphite and C_60_. Interestingly, the PDF patterns are almost identical in CMS and its heat-treated samples up to 2873 K. This means that CMS comprises a well-developed sp^2^-carbon network mainly consisting of carbon hexagons, and the number of non-hexagonal rings introduced by zipping reactions is insignificant. Indeed, the ratio of carbon polygons at the boundary of the graphene fragments in CMS (*ca.* 6.9 nm) is estimated to be as low as 7%. Note that not all the boundary sites become topological defects. Undetectable carbon 5- and 7-membered rings in Fig. 5c suggest that some of the boundaries form new carbon hexagons after coalescence.

The presence of the edge sites is further analyzed by H_2_O-vapor adsorption measurements, as shown in Fig. S5.† The amount of H_2_O adsorption at low pressure generally decreases with increasing the HT temperature. Since carbon materials are hydrophobic, H_2_O can be adsorbed by carbon edge-sites at low pressure,^18^ and thus, this change corresponds to the decrease of the carbon edge sites upon annealing.

### Change of other physicochemical properties by HT

The solid-state ^13^C NMR spectra of CMS before and after HT at 2073 K are shown in Fig. S6.† Both samples show a peak of around 115 ppm, typical in sp^2^-carbon materials, and are free from the peak of sp^3^-carbons around 40–60 ppm.^55,56^ These results suggest that the sp^2^-carbon network of the CMS framework is well developed and cannot be distinguished from the sample annealed at 2073 K by ^13^C NMR.

SXES provides information on the valence electron structures of carbon materials.^57^ As shown in Fig. S7,† HT of CMS up to 2873 K hardly changes the spectrum shape. The SXES results accord well with the results of PDF (Fig. 5) and ^13^C NMR: CMS already comprises a well-developed sp^2^-carbon network of carbon hexagons. However, as shown below, its magnetic properties, chemical and electrochemical stability, mechanical properties, and electric conductivity greatly differ from its heat-treated samples. This means that PDF, ^13^C NMR, and SXES cannot predict these properties of porous carbon materials.

**Fig. 6:**
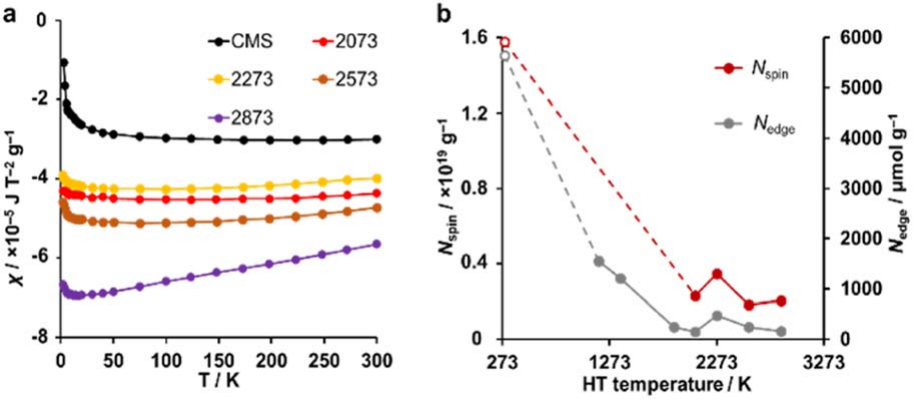
(a) The magnetic susceptibility for CMS and its heat-treated samples. (b) *N*_spin_ and *N*_edge_ plotted *versus* HT temperature. *N*_spin_ was calculated assuming *S* = 1/2. CMS is plotted at 298 K for a reference.

A magnetic susceptibility measurement was performed to determine the spin density. According to the Curie–Weiss law, the magnetic susceptibility from unpaired electron spins is inversely proportional to measurement temperature.^26,31^ As shown in Fig. 6a, CMS shows clear Curie paramagnetism in the region below 50 K, which is reduced in the samples after HT. In carbon materials, unpaired electrons are generally believed to exist at carbon edge sites.^58^ Therefore, the fusion of the edge sites by annealing should induce a decrease in the number of unpaired electron spins. Indeed, the annealing temperature dependence of the unpaired electron density (*N*_spin_) and *N*_edge_ measured by TPD show similar trends (Fig. 6b). In the high-temperature range of 50–300 K, the absolute value of magnetic susceptibility (*χ*) is generally enhanced with increasing annealing temperature. The sample annealed at 2873 K shows the largest *χ*, and the absolute value decreases with increasing the measurement temperature. This characteristic temperature dependence is ascribed to Landau diamagnetism originating from cyclotron motion of conduction π-electrons in the carbon basal plane.^59–61^ Thus, HT at high temperatures enhances the diamagnetism because of the increase in the graphene-domain size and graphene stacking.

### Oxidation resistance

**Fig. 7:**
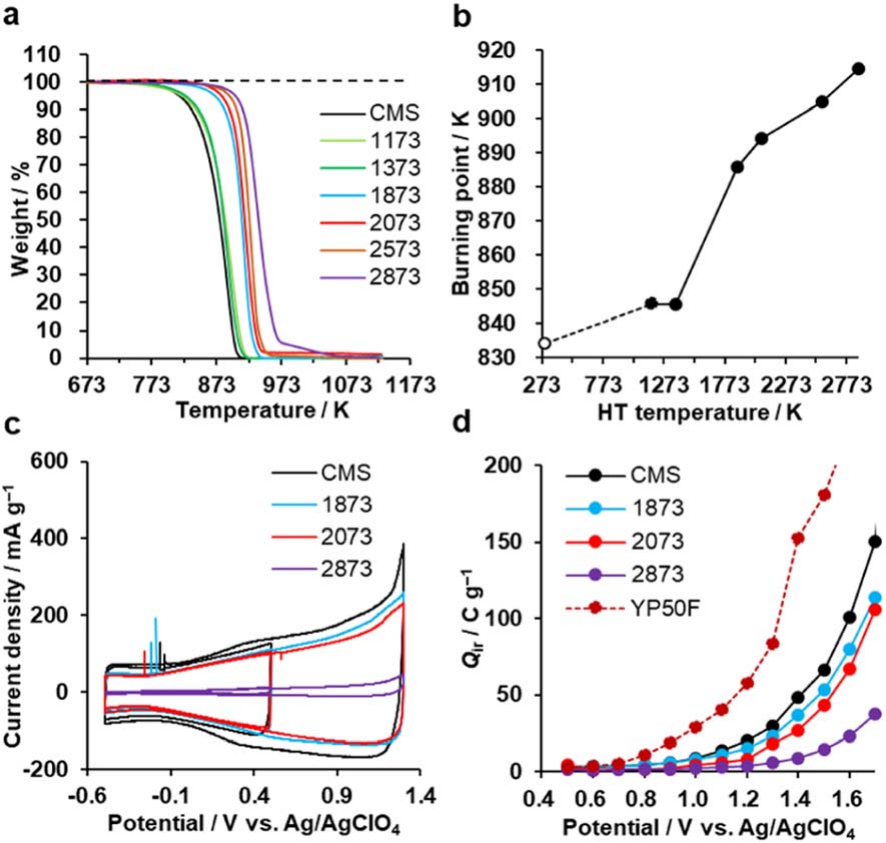
Oxidation resistance behaviours. (a) TG curves of CMS and its heat-treated samples and (b) burning point plotted *versus* HT temperature. CMS is plotted at 298 K for a reference. (c) CV patterns (1st cycle) of CMS and its heat-treated samples in a potential range of −0.5 to 0.5 V and −0.5 to 1.3 V. (d) *Q*_ir_ of CMS and its heat-treated samples, together with reference YP50F.

The oxidation resistance of CMS and its heat-treated samples were examined by thermogravimetry (TG) in air. We define the burning point as the temperature at which the weight loss reaches 10 wt% of the sample weight. In Fig. 7a and b, CMS is least resistant to oxidation due to its abundance of edge sites from which oxidative gasification can proceed. From 1373 to 1873 K, the oxidation resistance remarkably improves due to the significant decrease of edge sites, as found in Fig. 3d. In addition, further annealing above 2073 K also improves its oxidation resistance, indicating that the reduction of topological defects and the development of graphene stacking are effective in achieving a better oxidation resistance.

Next, the electro-oxidation resistance is examined by *Q*_ir,_ which is calculated from CV patterns shown in Fig. 7c and S8.†^35^ The electro-oxidation resistance is crucial for electrode applications to supercapacitors,^17^ polymer-electrolyte fuel cells,^22^ all-solid Li-sulfur batteries,^20^ and Li–O_2_ batteries.^24^ When *Q*_ir_ is suppressed even at a high potential, the material is stable.^35^ As shown in Fig. 7d, the stability is improved by increasing the HT temperature. This indicates that electro-oxidation resistance also depends on the number of edge sites, the number of topological defects, and the graphene stacking structures, like air-oxidation resistance. CMS and its derivatives all outperform the commercial activated carbon YP50F. Considering the balance of nanoporosity (Fig. S1†) and electro-oxidation resistance (Fig. 7d), we can conclude that HT between 1873 K and 2073 K is the most promising to improve the properties in highly mesoporous carbons consisting of a few-layer graphene wall.

### Flexibility

**Fig. 8:**
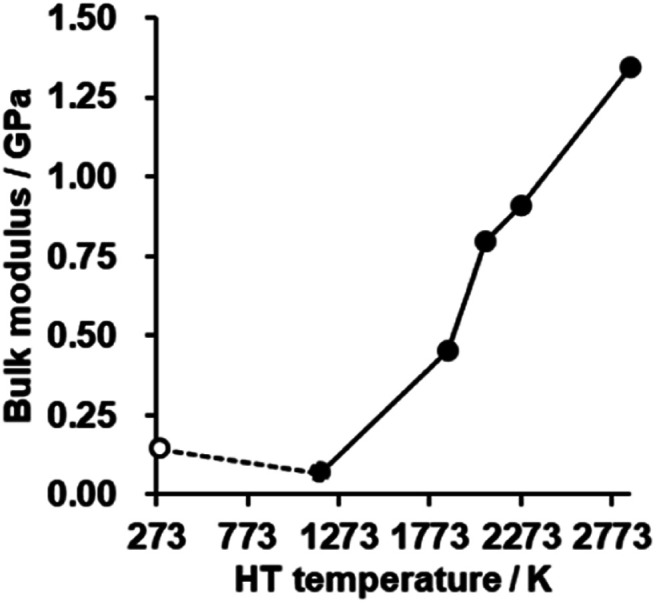
The bulk modulus of CMS and its heat-treated samples. CMS is plotted at 298 K for a reference.

The bulk modulus of a porous carbon material measured by a mercury intrusion test represents its resistance to changes in volume under pressure.^62^ The bulk modulus is plotted against HT temperature in Fig. 8 (for details, see also Fig. S9†). There is no significant difference in CMS before and after HT at 1173 K, but the bulk modulus is enhanced significantly with increasing the HT temperature. As analyzed above, the zipping reaction of edge sites completes at around 2073 K, and is then followed by the stacking of the graphene sheets. Thus, the marked increase in the bulk modulus below 2073 K can be attributed to the formation of the continuous graphene framework from the zipping reactions. In addition, the mesopores become harder upon annealing above 2073 K due to the increased stacking of graphene sheets. CMS and its heat-treated product at 2073 K exhibit relatively low bulk modulus compared to conventional nanoporous carbons,^19^ which means they are mechanically soft. These kinds of flexible nanoporous materials are expected to have great potential for expandable adsorbents^18^ and new types of heat pumps.^19^

### Electrical conductivity

**Fig. 9:**
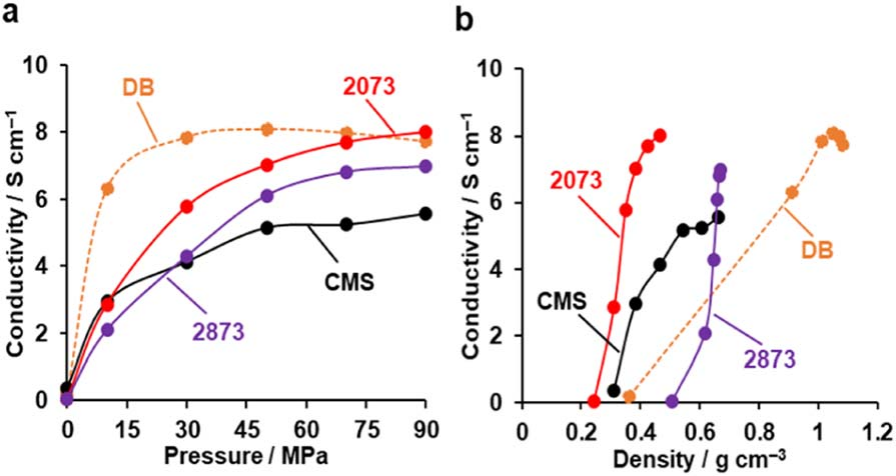
The electric conductivity of powdery CMS, its heat-treated samples, and reference DB. (a) Conductivity *versus* pressure. (b) Conductivity *versus* powder density.

Fig. 9a shows the electrical conductivities of powdery CMS, its heat-treated samples, and DB (denka black, a type of commercial carbon black, used as a reference) measured by a two-probe method.^63^ The conductivity of CMS increases significantly upon HT at 2073 K, which can be attributed to the reduction of edge sites and the formation of continuous graphene sheets. Note that compressing the powder sample leads to changes in density and the resistance between the sample particles. From Fig. 9b, the density of DB is sensitive to pressure. When comparing the conductivity at the same density, both CMS before and after HT at 2073 K exhibit a higher conductivity than DB. On the other hand, HT at 2873 K makes the sample mechanically hard (Fig. 8), and much higher pressure is required to increase the sample-bed density. Furthermore, the stacked graphene structure reduces conductivity due to intralayer coupling and scattering.^64^

The comparison of the oxidation resistance, elasticity, and electric conductivity performance of CMS and its heat-treated samples, these performances are highly related to the size of graphene sheets, the concentration of carbon edge sites, and the number of stacked graphene sheets. As the HT temperature increases, the degree of crystallinity increases, and the number of carbon edge sites decreases, resulting in improved electrical conductivity and chemical stability; however, a decrease in flexibility is also observed. In addition, graphene layer stacking is accompanied by HT above 2073 K, which is unfavorable for its specific surface area, flexibility, and sample-bed-based electrical conductivity.

## Conclusions

We analyzed a series of mesoporous carbons (CMS, and its heat-treated samples between 1173 K and 2873 K) with a graphene wall comprised of 1–2 graphene layers. Our results show that the well-developed sp^2^-carbon network and the mesopore structure remain intact until 2073 K. At higher temperatures, pores collapse and graphene layer stacking occurs, accompanied by morphological changes and increased crystallization. Carbon edge sites, where oxidation initiates, are mostly removed at 1873 K, and a larger continuous graphene framework is formed as the result of graphene-zipping reactions that take place between neighboring graphene domains. Oxidation resistance, elasticity, and electrical conductivity are strongly related to the number of edge sites, topological defects, the size of the continuous graphene sheet, and the number of layers in the graphene structures. Therefore, HT at 1873–2073 K can achieve excellent electrical conductivity, high durability, and unique mechanical flexibility due to the minimal number of edge sites, large continuous graphene sheet, and still intact mesoporous structure without graphene layer stacking. Benefiting from these unique properties, it exhibits excellent potential on supercapacitors, lithium batteries, polymer-electrolyte fuel cells, catalyst supports, high-capacity adsorbents, and new types of heat pumps. These findings provide a reliable and systematic study that contributes to the understanding and advancement of the synthesis and applications of porous carbon materials with 1273 K to 2273 K heat treatment, as well as the controllable design of other carbonaceous frameworks with desired properties.

## Data availability

All data supporting the findings of this study are available from the corresponding author upon reasonable request.†

## Author contributions

T. X., T. Y., K. N., and H. N. designed the study, performed the experiments, and analyzed the data. T. M. and J. M. contributed to the sample preparation. H. T. performed the molecular dynamics simulation. T. O. and K. I. performed the neutron diffraction experiments. Y. S. and M. T. performed the SXES experiments. K. W. and T. I. contributed to the SQUID experiment and data analysis. Z. P. contributed to the data analysis of mercury intrusion and electric conductivity measurements. T. X., T. Y., and H. N. wrote the manuscript. T. Y. and H. N. supervised the project. T. K. helped supervise the project. All authors have given approval to the final version of the manuscript.

## Conflicts of interest

There are no conflicts to declare.

## Supplementary Material

SC-014-D3SC02163G-s001
