# Supplementary material for: Chemistry of zipping reactions in mesoporous carbon consisting of minimally stacked graphene layers
Source: Chem Sci. 2023 Jul 18;14(32):8448–57. doi: 10.1039/d3sc02163g (PMC10430703; doi:10.1039/d3sc02163g)
Supplement: SC-014-D3SC02163G-s001 [file SC-014-D3SC02163G-s001.pdf]

Electronic Supplementary Material (ESI) for Chemical Science.
This journal is © The Royal Society of Chemistry 2023

Supporting Information

Chemistry of zipping reactions in mesoporous carbon consisting of minimally stacked graphene layers

Tian Xia,[a] Takeharu Yoshii,*[a] Keita Nomura,[a] Keigo Wakabayashi,[a] Zheng-Ze Pan,[b] Takafumi Ishii,[c] Hideki Tanaka,[d] Takashi Mashio,[e] Jin Miyawaki,[e,f] Toshiya Otomo,[g,h,i,j] Kazutaka Ikeda,[g,h,i] Yohei Sato,[a] Masami Terauchi,[a] Takashi Kyotani,[a] Hirotomo Nishihara *[a,b]

[a] Institute of Multidisciplinary Research for Advanced Materials, Tohoku University, 2-1-1 Katahira, Aoba-ku, Sendai, Miyagi, 980-8577, Japan

[b] Advanced Institute for Materials Research, Tohoku University, 2-1-1 Katahira, Aobaku, Sendai, Miyagi, 980-8577, Japan

[c] International Research and Education Center for Element Science Faculty of Science and Technology, Gunma University, 1-5-1 Tenjincho, Kiryu, Gunma, 376-8515, Japan

[d] Research Initiative for Supra-Materials (RISM), Shinshu University, 4-17-1 Wakasato, Nagano, 380-8553, Japan

[e] Interdisciplinary Graduate School of Engineering Sciences, Kyushu University, 6-1 Kasuga-koen, Kasuga, Fukuoka, 816-8580, Japan

[f] Institute for Materials Chemistry and Engineering, Kyushu University, 6-1 Kasuga-koen, Kasuga, Fukuoka, 816-8580, Japan

[g] Institute of Materials Structure Science, High Energy Accelerator Research Organization (KEK), 203-1 Shirakata, Tokai, Ibaraki, 319-1106, Japan

[h] J-PARC Center, High Energy Accelerator Research Organization (KEK), 2-4 Shirakata-Shirane, Tokai, Ibaraki, 319-1106, Japan

[i] School of High Energy Accelerator Science, The Graduate University for Advanced Studies, 203-1 Shirakata, Tokai, Ibaraki, 319-1106, Japan

[j] Graduate School of Science and Engineering, Ibaraki University, 162-1 Shirakata, Tokai, Ibaraki, 319-1106, Japan

Corresponding Author

*Email: takeharu.yoshii.b3@tohoku.ac.jp, hirotomo.nishihara.b1@tohoku.ac.jp

N_2 adsorption-desorption isotherms

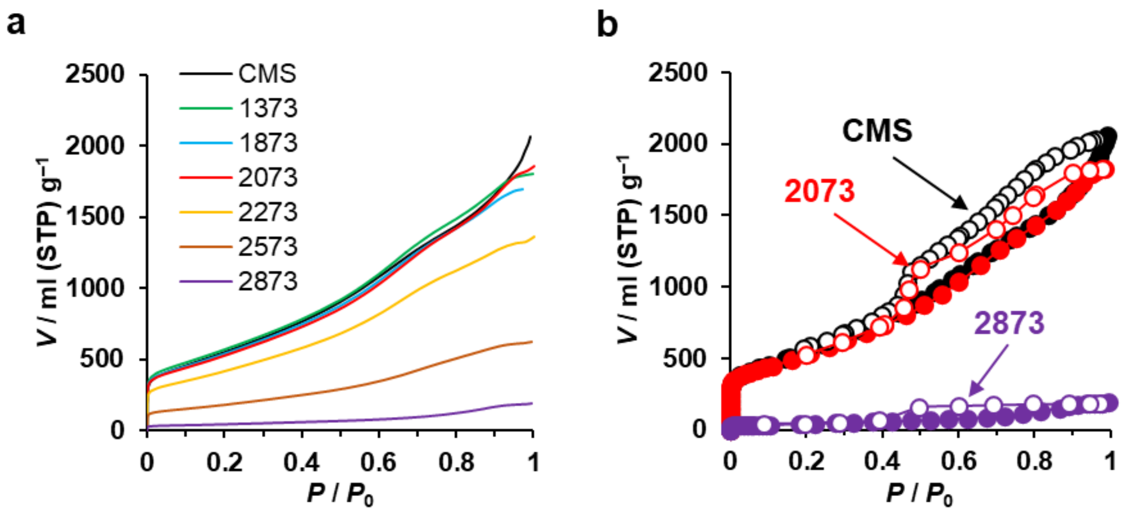

Figure S1 (a) N_2 adsorption isotherms of CMS and its heat-treated samples. (b) N_2 adsorption-desorption isotherms of CMS and samples heat-treated at 2073 and 2873 K.

PXRD and Raman results

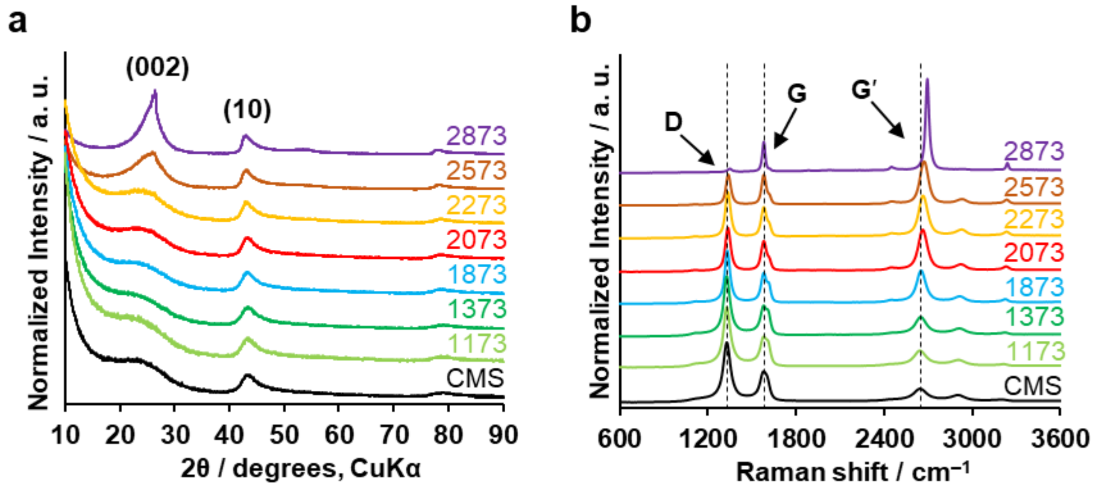

Figure S2 (a) PXRD patterns and (b) Raman spectra of CMS and its heat-treated samples. The peak positions of D-, G-, and G'-bands of CMS are shown with dashed vertical lines.

TPD results

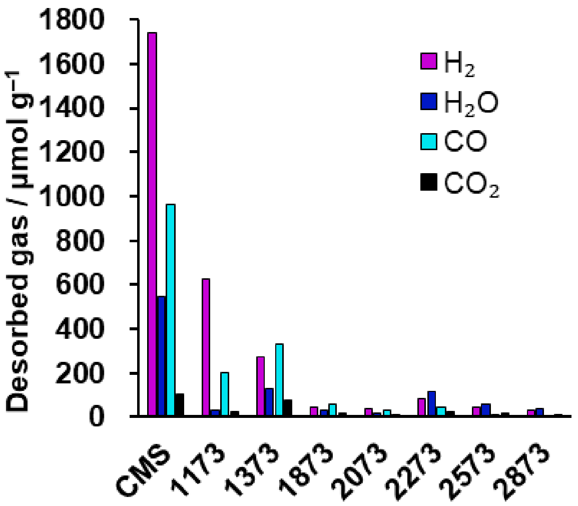

Figure S3 Desorbed amounts of H_2, H_2O, CO and CO_2 from the TPD analysis of CMS and its heat-treated samples.

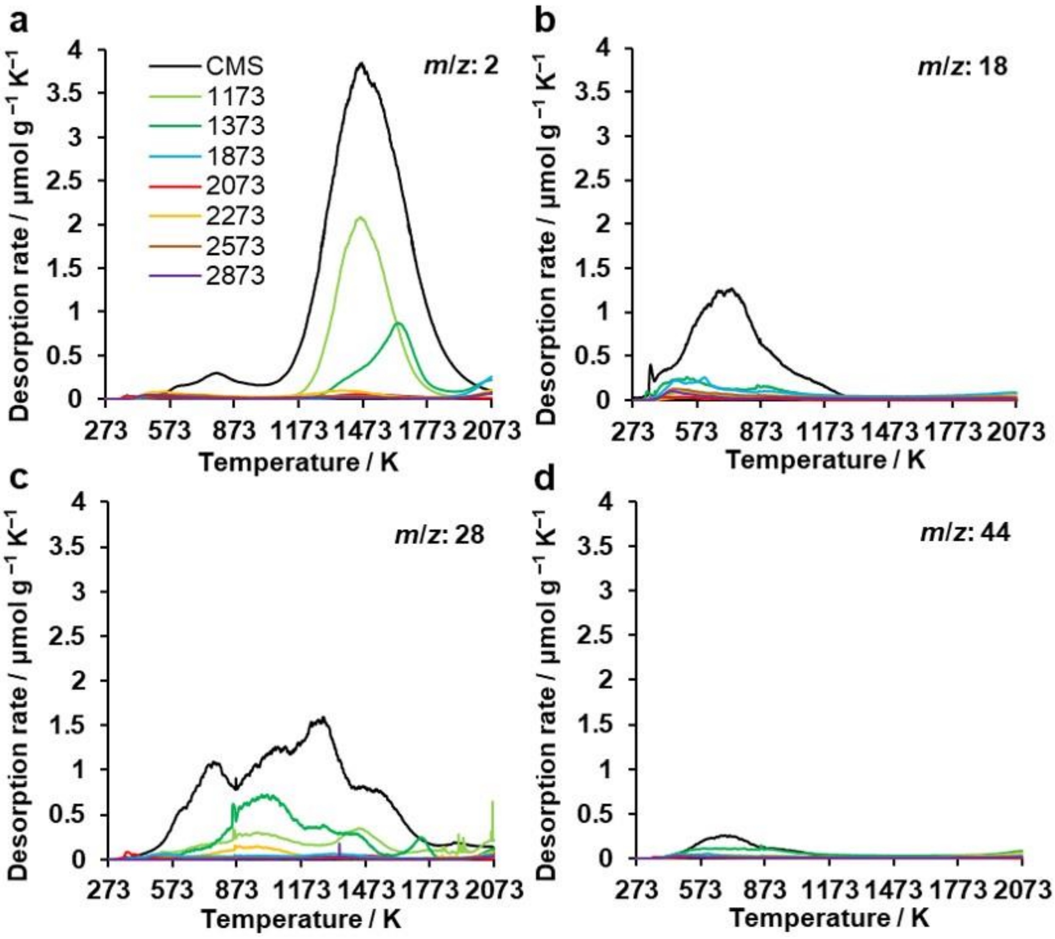

Figure S4 The gas-evolution profiles of CMS and its heat-treated samples for a) H_2 (m/z = 2), b) H_2O (m/z = 18), c) CO (m/z = 28), and CO_2 (m/z = 44).

H_2O-vapor adsorption

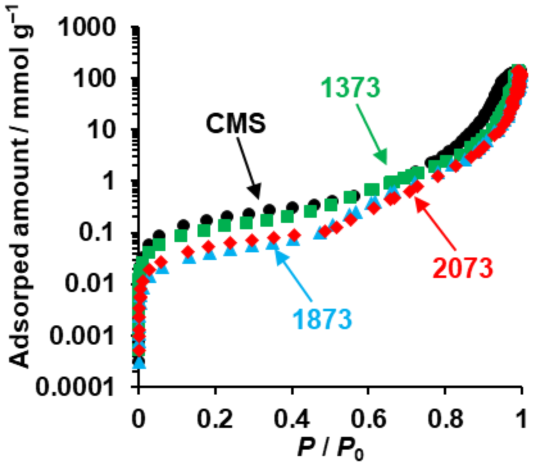

Figure S5 H_2O-vapor adsorption isotherms on CMS and its heat-treated samples at 298 K.

Solid-state ^{13}C NMR

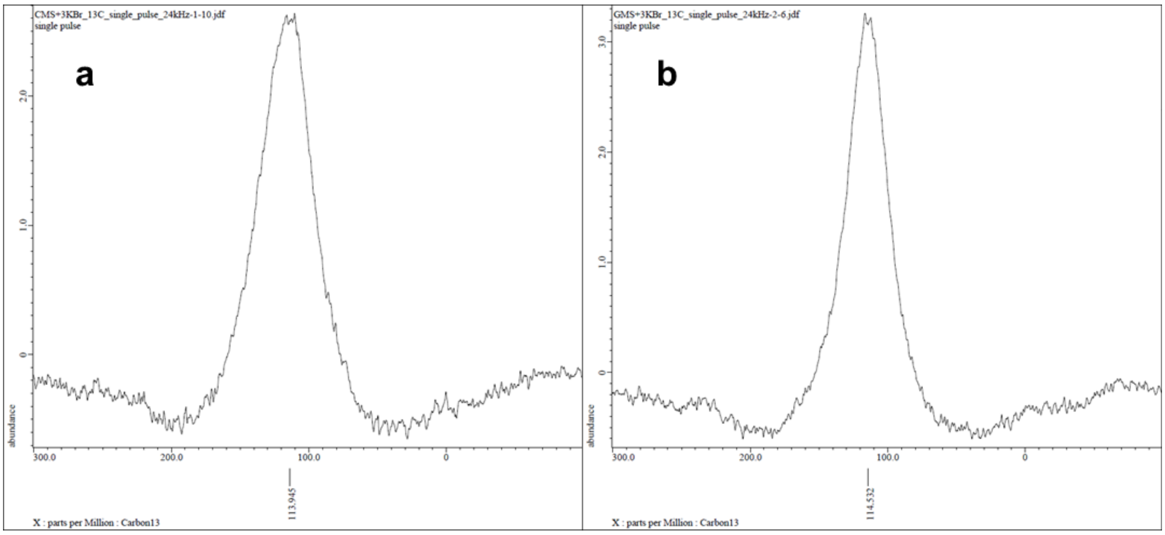

Figure S6 Solid-state ^{13}C NMR spectra of a) CMS and b) CMS treated at 2073 K.

SXES

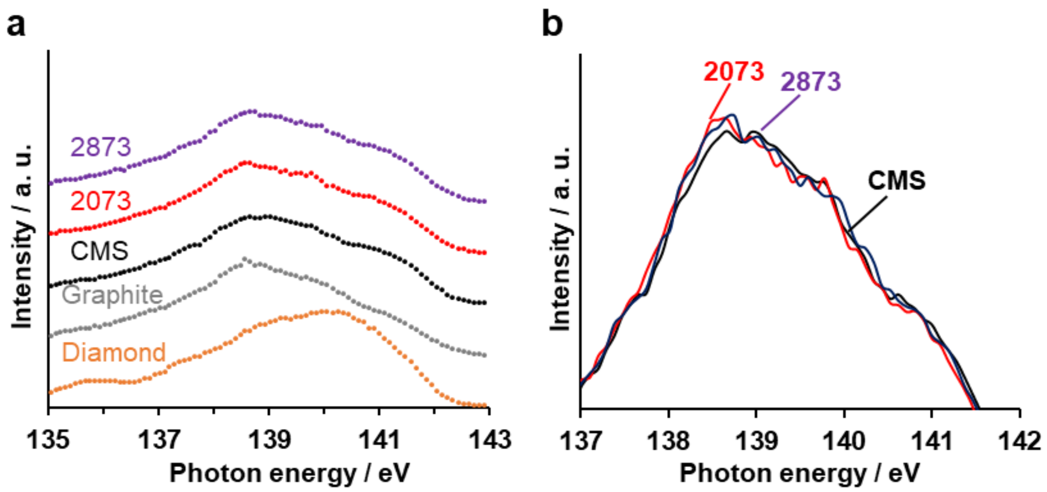

Figure S7 (a) Soft X-ray emission spectra (SXES) of CMS, its heat-treated samples, graphite, and diamond. (b) Enlarged patterns for CMS and its heat-treated samples.

CV results

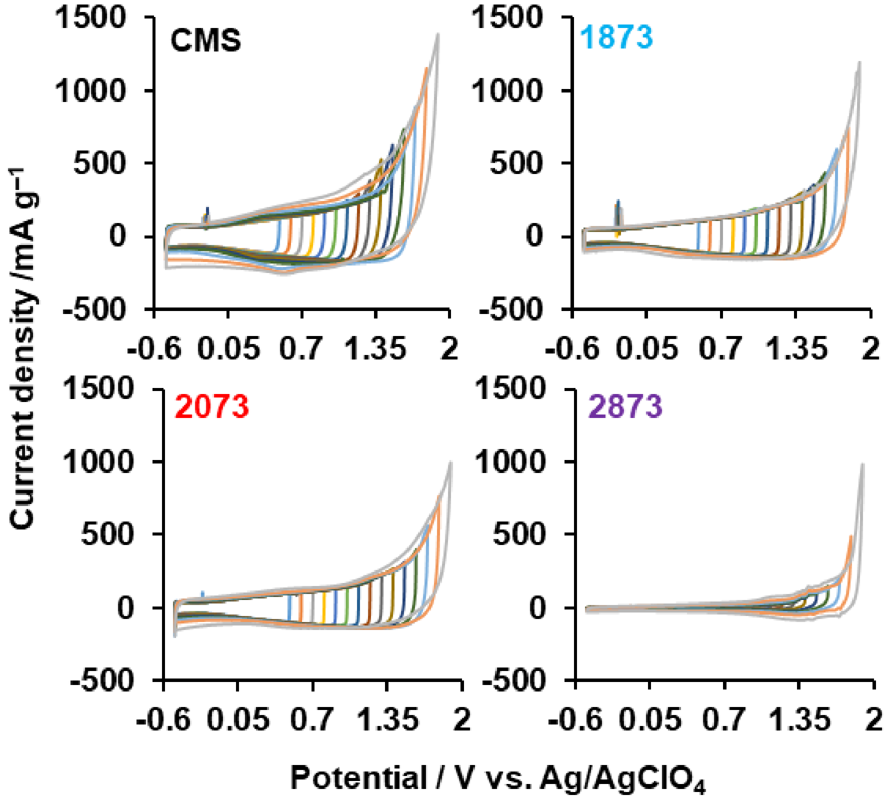

Figure S8 CV curves of CMS and its heat-treated samples measured with a three-electrode cell in 1M Et_4NBF_4/PC at 298 K. The first cycle at each potential range is shown. The scan rate is 1 mV s^{-1}. The CV curves shown here were used for the calculation of Q_{ir}.

Mercury intrusion

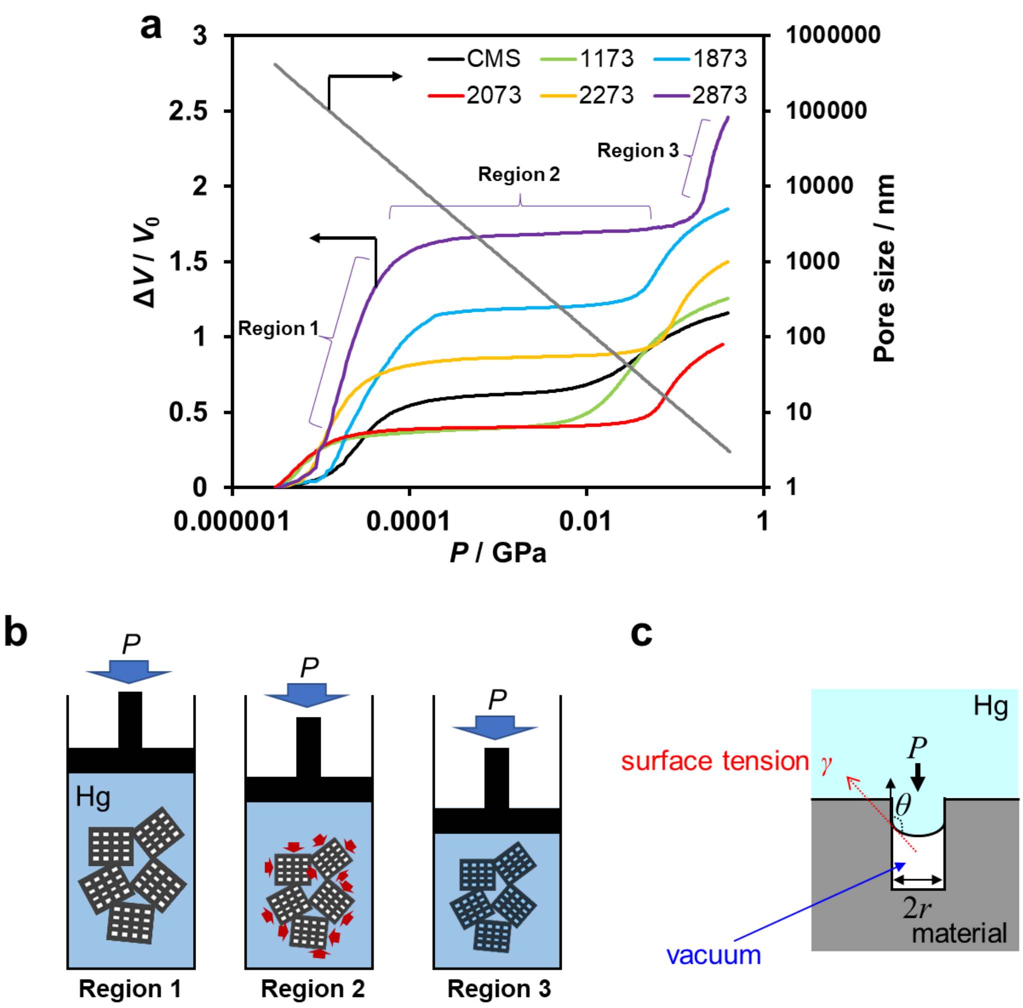

Figure S9 Mercury intrusion results of CMS and its heat-treated samples. (a) Applied pressure against $\Delta V/V_0$. ΔV and V_0 are the volume change of mercury+sample and initial sample volume, respectively. The relationship between the mercury pressure and the pore size which is filled with Hg is plotted as a straight gray line. (b) The illustration of explanation of principle of mercury intrusion test. (c) The illustration of contact between mercury and the porous material, in which the following equation holds

$$r = -\frac{2\gamma \cos\theta}{P}$$

where r, P_{Hg}, γ, and ϑ are the radius of the capillary, the pressure of Hg, the surface tension of Hg, and the contact angle of Hg.

Table S1 Comparison of L_{PXRD} and L_{TPD} of CMS and its heat-treated samples.

	**CMS**	**1173**	**1373**	**1873**	**2073**	**2873**
L_{PXRD} [nm]	6.2	6.4	7.1	7.5	8.2	12.5
L_{TPD} [nm]	6.9	26.2	33.4	165	270	260
